# Supplementary material for: Physical Activity versus Selected Health Behaviors, Subjective Physical and Psychological Health and Multimorbidity in a Large Cohort of Polish Seniors during the COVID-19 Pandemic (Results of the National Test for Poles’ Health )
Source: Int J Environ Res Public Health. 2022 Dec 29;20(1):556. doi: 10.3390/ijerph20010556 (PMC9819469; doi:10.3390/ijerph20010556)
Supplement: Supplementary file 1 [file ijerph-20-00556-s001.zip › NTZP Questionnaire.pdf]

NTZP Questionnaire – selected questions for the purpose of analysis

Physical activity versus selected health behaviors, subjective physical and psychological health and multimorbidity in a large cohort of Polish Seniors during COVID-19 pandemics (results of the National Test for Poles' Health).

Q1. Please select your gender:

1 Woman

2 Man

Q2. Please enter your age.

Q3. What education have you received so far?

1 Primary

2 Junior high school

3Vvocational

4 Secondary

5 Post-secondary

6 Bachelor / Engineer

7 Master's degree

Q4. Please select the size of the town where you currently live.

1 village

2 city up to 19 thousand residents

3 city 20 - 49 thousand residents

4 city 50 - 99 thousand residents

5 city 100 - 199 thousand residents

6 city 200 - 499 thousand residents

7 city 500 thousand or more residents

Q6. Please enter your height in centimeters.

Q7. Please enter your body weight (weight) in kilograms.

Q8. How do you generally evaluate your physical health compared to other people of your age?

1 Very good

2 Good

3 Average (neither good nor bad)

4 Bad

5 Very bad

Q9. How do you generally evaluate your mental health compared to other people of your age?

1 Very good

2 Good

3 Average (neither good nor bad)

4 Bad

5 Very bad

Q10. Do you have any long-term health problems or chronic illnesses that last (or are expected to last) 6 months or more?

1 Yes

2 No

Q11. Have you been diagnosed with any of the following diseases / conditions?

1 - YES 2 - NO

1 Hypertension

2 Diabetes

3 Heart disease (ischemic heart disease, heart valve defect, heart failure, atrial fibrillation)

4 Chronic Obstructive Pulmonary Disease (COPD)

5 Depression

6 Cancer

7 Obesity

Q46. On average, how much time a day do you spend on walking activities - as a form of recreation and rest or as a form of reaching your destination (work, home, etc.)?

1 Up to 30 minutes

2 30 to 60 minutes

3 1 to 2 hours

4 More than 2 hours

5 I don't leave the house / walk for health reasons

6 I don't leave the house / walk for other reasons

Q47. How much time do you spend on average on engaging in sports?

1 At least 2 hours a week or more

2 About 1-1.5 hours a week

3 Less than 4 hours a month

4 I don't do any sports
